# Presenting the COGNIFOG Framework: Architecture, Building Blocks and Road toward Cognitive Connectivity

**DOI:** 10.3390/s24165283

**Published:** 2024-08-15

**Authors:** Toni Adame, Emna Amri, Grigoris Antonopoulos, Selma Azaiez, Alexandre Berne, Juan Sebastian Camargo, Harry Kakoulidis, Sofia Kleisarchaki, Alberto Llamedo, Marios Prasinos, Kyriaki Psara, Klym Shumaiev

**Affiliations:** 1 Fundació i2CAT, Gran Capità 2-4, 08034 Barcelona, Spain; juan.camargo@i2cat.net; 2 CYSEC SA, EPFL Innovation Park Batiment A, 1015 Lausanne, Switzerland; emna.amri@cysec.com; 3 Netcompany-Intrasoft, Fragkokklisias 13, 15125 Maroussi, Greece; grigoris.antonopoulos@netcompany.com; 4 Commissariat à l’Énergie Atomique et aux Énergies Alternatives, Rue Leblanc 25, 75015 Paris, France; selma.azaiez@cea.fr (S.A.); alexandre.berne@cea.fr (A.B.); 5 Telematic Medical Applications, Skra 1-3, 17673 Kallithea, Greece; hariskakoulidis@tma.gr (H.K.); mprasinos@tma.gr (M.P.); 6 Kentyou, Cours Berriat 93, 38000 Grenoble, France; sofia.kleisarchaki@kentyou.com; 7 ATOS IT, Ronda de Europa 5, 28760 Tres Cantos, Spain; alberto.llamedo@eviden.com; 8 eBOS Technologies Limited, Arch. Makariou III and Mesaorias 1, Lakatamia 2322, Cyprus; kyriakip@ebos.com.cy; 9 Siemens Aktiengesellschaft, Werner-von-Siemens-Straße 1, 80333 Munich, Germany; klym.shumaiev@siemens.com

**Keywords:** IoT-edge-cloud continuum, service orchestration, cognitive connectivity, fog computing, edge computing, IoT, intelligent systems, confidential computing

## Abstract

In the era of ubiquitous computing, the challenges imposed by the increasing demand for real-time data processing, security, and energy efficiency call for innovative solutions. The emergence of fog computing has provided a promising paradigm to address these challenges by bringing computational resources closer to data sources. Despite its advantages, the fog computing characteristics pose challenges in heterogeneous environments in terms of resource allocation and management, provisioning, security, and connectivity, among others. This paper introduces COGNIFOG, a novel cognitive fog framework currently under development, which was designed to leverage intelligent, decentralized decision-making processes, machine learning algorithms, and distributed computing principles to enable the autonomous operation, adaptability, and scalability across the IoT–edge–cloud continuum. By integrating cognitive capabilities, COGNIFOG is expected to increase the efficiency and reliability of next-generation computing environments, potentially providing a seamless bridge between the physical and digital worlds. Preliminary experimental results with a limited set of connectivity-related COGNIFOG building blocks show promising improvements in network resource utilization in a real-world-based IoT scenario. Overall, this work paves the way for further developments on the framework, which are aimed at making it more intelligent, resilient, and aligned with the ever-evolving demands of next-generation computing environments.

## 1. Introduction

In the ever-evolving technological landscape, marked by the omnipresence of the Internet and the unstoppable digitalization of processes and services, the consolidation of next-generation technology drivers, such as Artificial Intelligence (AI), the Internet of Things (IoT), and cloud computing, have opened up new possibilities to address current and future challenges across the fabric of our daily lives. However, this progress has also brought to the forefront the challenge of effectively managing the massive data generated by the resulting new systems.

The current volume of data generated in the Internet is truly astonishing: in 2020 alone, the total amount of data created, captured, copied, and consumed globally was 64 trillion gigabytes [1], and that value is accelerating due to the growing adoption of IoT applications and services in various domains with a notable emphasis on home and industrial automation, healthcare, agriculture, and transportation. In fact, by the end of 2022, the number of worldwide active IoT devices reached 13.2 billion, which is a figure expected to increase to 34.4 billion by 2032 [2].

Most existing IoT architectures exhibit high centralization, heavily relying on the transfer of data processing, analytics, and decision-making processes to cloud solutions. While conceptually straightforward, cloud computing can lead to inefficiencies related to latency, network traffic management, computational processing, and power consumption [3]. Additionally, centralized IoT architectures struggle to meet the stringent requirements of cutting-edge time-sensitive applications whose performance and timeliness are compromised by the delays introduced when transferring data to the cloud and then back to on-site actuators [4].

Conversely, edge computing promotes the processing of data close to where it is generated in the physical world. By means of several techniques such as data fusion, trend analysis, and/or partial decision making, it is possible to minimize the amount of data being sent to the cloud. As a result, edge computing effectively tackles challenges related to network traffic, bandwidth (BW) consumption, latency, and overall energy usage. Moreover, edge distributed infrastructure contributes to enhanced resilience in the event of a significant incident (such as a large-scale cyberattack) by ensuring local resources and services remain accessible offline. Additionally, the fog computing concept was first introduced by Cisco Systems in 2012 [5], extending the edge computing domain by introducing an additional layer of distributed computing resources between the edge and the cloud.

The wide ecosystem where IoT devices and the presented architectures converge to form a computing continuum is known as the IoT–edge–cloud continuum [6]. This approach extends the high-performance cloud data centers with energy-efficient and low-latency devices close to the data sources [7]. Typical workloads from AI-based data analytics are perfect candidates for this continuum, since raw data are generated by IoT devices, whereas data-intensive analysis is performed on centralized locations in the cloud [8]. For their part, edge servers operate as an extension of the centralized cloud in the context of an overall application that is managed in the cloud but executed at the edge.

One of the main challenges of the IoT–edge–cloud continuum is the effective orchestration of distributed resources in a heterogeneous ecosystem; that is, the flexible and dynamic provisioning and management of computing, storage, and networking resources while ensuring the security and sovereignty of data. In fact, IoT applications can greatly benefit from smart orchestration to reduce energy consumption, overall latency, and message overload as well as achieve more efficient data storage and data transmission rate in large-scale scenarios. However, it involves a complex interplay of computational resources, learning models, algorithms, networking capabilities, and edge/IoT device mobility patterns [9].

The COGNIFOG framework introduced in this paper represents an innovative cognitive fog solution tailored to meet the challenges of next-generation computing. Currently in development, it carries the name of the Horizon Europe project COGNIFOG, which will conclude by the end of 2025 [10]. This framework aims to enable the seamless orchestration of diverse distributed computing resources across various IT systems, integrating IoT, edge, and cloud ecosystems. By creating a unified fog continuum, COGNIFOG aspires to achieve the following:

- Minimize energy consumption and latency by shifting data processing to the edge instead of routing it through communication networks to a data center.
- Cut operational expenses and accelerate service delivery by dynamically provisioning computing, storage, and networking resources with minimal human oversight.
- Enable the rapid development and deployment of applications by means of open application programming interfaces (APIs).

The remainder of this document is organized as follows: Section 2 reviews the current state of the art in IoT–edge–cloud continuum systems and their main challenges. Section 3 presents the architecture, building blocks, and main features of the COGNIFOG framework. The discussion in Section 4 then focuses on connectivity challenges, demonstrating the potential of COGNIFOG to efficiently manage network resources. Section 5 incorporates a preliminary implementation of COGNIFOG in an IoT testbed environment and assesses its benefits in terms of connectivity performance. Lastly, conclusions and future work are discussed in Section 6.

## 2. State of the Art

The convergence of technologies such as edge–fog–cloud computing, AI, and IoT into a real AI-empowered IoT–edge–cloud continuum for self-aware cognitive computing environments is highly likely to create opportunities for creative applications and innovative business models in sectors ranging from healthcare to Industry 4.0 [11]. However, their full potential is yet to be realized, as their effective integration faces several substantial challenges [12]. The following subsections delve into some of these challenges, offering insights and potential solutions.

### 2.1. IoT–Edge–Cloud Continuum Frameworks

Numerous initiatives are addressing global communication and management protocols within the IoT–edge–cloud continuum, aiming to establish a unified data management system that supports multiple heterogeneous services, implements appropriate security mechanisms, and ensures optimal resource allocation. However, to date, there is a notable absence of a unified open-source framework or a reference standard that facilitates comprehensive operations across large-scale infrastructures [13]. In the following section, to the best of our knowledge, we will briefly review some of the most significant initiatives.

The UNITE architecture, presented in [14], stands for integrating computing, networking, and storage resources across the IoT–edge–cloud continuum. This architecture enables end users to utilize all the aforementioned resources in a seamless and straightforward manner. Furthermore, it conducts ongoing resource monitoring and employs AI methods to predict and proactively address potential issues, such as network congestion and cloud server overload.

An innovative platform-as-a-service (PaaS) is proposed in [15] to facilitate the dynamic provisioning and management of cloud-native, multi-access edge computing (MEC)-based IoT applications across hybrid clouds. Leveraging open source projects from the Cloud Native Computing Foundation (CNCF) [16] and the Apache Software Foundation [17,18], the described architecture simplifies development and orchestrates data-intensive, intelligence-driven IoT applications, ensuring security and privacy.

To enhance the self-management of the IoT–edge–cloud continuum, the authors of [19] introduce the concept of the *intent-based* cognitive continuum, where the possible intents encompass constraints, preferences, expectations, goals, the type of entities that are relevant to these intents, and the relationships among them. By means of AI techniques, a unified data layer, and intent-based human–AI interaction, their approach addresses limitations in cloud resource optimization. This contributes to sustainable elasticity by providing a high-level means to express application performance requirements.

In the simulation domain, an osmotic computing environment across an edge–cloud continuum is introduced in [20]. The authors characterize osmotic computing as the dynamic migration of workload between a cloud data center and edge devices, which is triggered by performance and security events. Consequently, they successfully model and simulate an IoT application centered around electricity management and billing within a smart city, operating over a heterogeneous edge–cloud environment.

The research in [21] proposes an optimized IoT-enabled big data analytics architecture for edge–cloud computing using machine learning (ML). Its two-layered scheme, combining IoT-edge and cloud processing, employs advanced algorithms for efficient data injection, processing, and storage, thus enhancing cluster management and addressing structural challenges in handling massive and heterogeneous data from IoT devices.

Existing frameworks often overlook the reality that certain constrained IoT devices lack the capacity to support specific tools. On this matter, RAMOS constitutes a novel reference architecture for a meta-OS designed to address the limitations of current IoT–edge–cloud infrastructures by facilitating a dynamic, distributed, and trusted continuum for data-intensive and ML-based applications [22].

Lastly, for a comprehensive review on further frameworks for the IoT–edge–cloud continuum, we refer the reader to [13,23].

### 2.2. Container-Based Orchestration

Fog computing has effectively expanded traditional cloud computational resources and services to the network edge, improving proximity to user devices and reducing latency. Unlike cloud computing, fog and edge resources may rely on resource-restricted devices that cannot cope with the deployment of conventional virtual machines (VMs) to run different environments simultaneously.

Containers instead are known for their lightweight and small footprint virtualization and provide a consistent, secure, and portable environment for running software across heterogeneous computing environments. Additionally, the growing popularity of microservices, serving as a substitute for traditional monolithic software architectures, has notably accelerated the adoption of container-based virtualization. Due to microservices, software applications can now be decomposed into smaller components that can be managed independently. Despite the fact that microservices can be deployed in both VMs and containers, the latter offer higher scalability, flexibility, and agility [24].

In large, dynamic microservice platforms, containers are organized into clusters. Specifically, a cluster consists of a set of node machines intended to run containerized applications. Such a cluster contains a control plane and one or more computing nodes, whether physical or virtual, located on premises or in the cloud; whereas the control plane is responsible for maintaining the desired state of the cluster, nodes actually run the applications and workloads.

As organizations increasingly embrace modern technologies to facilitate container usage, they encounter complex challenges in meeting the service level agreement (SLA) and quality of service (QoS) requirements. Achieving these goals then calls for orchestration solutions able to provision, deploy, scale, manage, and secure containerized applications without worrying about the underlying infrastructure. Moreover, it is expected that all these tasks are accomplished automatically, without human intervention, aiming for the paradigm of comprehensive self-healing systems.

In the context of the IoT–edge–cloud continuum, this task becomes even more complex as it strives to maintain application portability, scalability, and resilience within geographically distributed and heterogeneous clusters, sometimes even facing unstable connectivity and node availability [25]. Multi-cluster orchestration intends to extend the orchestration paradigm to manage applications and services across multiple clusters, spanning on-premises, hybrid, and multi-cloud environments while mitigating complexity, enhancing scalability, ensuring resilience, and bolstering security and compliance.

Some multi-cluster orchestration tools, such as Rancher Fleet [26], Open Cluster Management [27], and OpenShift [28], are currently being used in the continuum to achieve comprehensive communication and management of resources [29]. However, they often fail when dealing with common issues related to communication, networking, high availability, and service discovery, since they have not been completely adapted to the particularities of fog/edge computing [30].

Alternatively, some of the latest approaches to the multi-cluster orchestration paradigm have emerged as hierarchical edge orchestration frameworks, such as Oakestra [31], where resource constraints, dynamic infrastructures, and proximity requirements pose unique challenges. Oakestra adapts to edge environments by offering federated cluster management, delegated task scheduling, and semantic overlay networking. ML-based orchestration approaches have also been recently explored, where different learning techniques lead to dynamic workload allocation, predictive scaling, and anomaly detection [32].

### 2.3. Monitoring and Analytical Capabilities

The idea of an integrated IoT–edge–cloud continuum, embracing autonomous and intelligent systems, is intimately connected to the independent and self-reliant features of its network elements [33]. In this context, monitoring emerges as the primary instrument necessary for endowing the system with enhanced self-diagnostic and self-awareness capabilities. Following this, AI-driven analytical resources play a crucial role in fostering the system’s self-learning and self-optimization processes.

Monitoring tools play a crucial role in maintaining system health by gathering a range of metrics at both the infrastructure and service levels that will be later employed by the orchestration engine. The specific metrics that need to be monitored are determined by the resource requirements of the workload and the ability of the worker nodes to maintain resource isolation especially when faced with disruptions [34].

While monitoring in monolithic applications tends to be relatively straightforward and deterministic, the situation is markedly more complex when dealing with microservices. In fact, their intricate web of interconnections significantly complicates the process of gaining meaningful insights into a system’s internal state solely through the analysis of its external outputs. This complexity underscores the need for advanced monitoring strategies that can effectively navigate and interpret the multifaceted interactions within these systems [35].

Some of the most advanced and used tools for multi-cluster monitoring include the use of federated time-series databases such as Prometheus [36], whose federation capabilities allow for multiple instances, where metrics for each cluster are independently scraped to be later aggregated into a centralized monitoring system. OpenTelemetry [37] is an observability framework that provides a standardized way to collect telemetry data in a multi-cluster manner, enabling consistent instrumentation across different environments such as those present in the IoT–edge–cloud continuum.

### 2.4. Formal Methods for the Design of the IoT–Edge–Cloud Continuum

Formal methods help engineers to design and deploy applications correctly from the start, ensuring safety, performance, and timing guarantees. Applying these methods in the context of the IoT–edge–cloud continuum faces some challenges. The model should take into account the new architectural concepts composed by IoT, edge, and cloud nodes with different hardware capacities that use heterogeneous communication in a hyper-distributed environment with different timing constraints. The challenge here is to provide a method agnostic to all interoperability tools, OS, virtualization, and orchestration systems, while capturing necessary behavioral properties such as liveness, consistency, and real-time guarantees.

The synchronous data flow (SDF) is a model of computation and communication (MoCC) used for describing the behavior of digital systems [38]. It has been used in various fields such as signal processing, multimedia, and embedded systems design. An extension of the SDF model, called PolyGraph [39], is used here as a basis for a methodology to model, analyze, deploy, and monitor applications that will run in the continuum. PolyGraph proposes a high-level language and a set of software tools to model and analyze the application at design time. It also provides a code generator as well as a monitor generator. Monitors are included all along the data path generated from the specified models, so that safety and security are guaranteed.

### 2.5. Multi-Level Interoperability

In the rapidly evolving landscape of the IoT, heterogeneous wireless communication technologies have emerged to facilitate seamless connectivity among diverse devices and networks [40]. Prominent among these technologies are those that enable wireless personal area networks (WPANs) like Bluetooth low energy (BLE), those based on the IEEE 802.15.4 standard [41] (e.g., Zigbee, Thread, and 6LoWPAN), those based on the IEEE 802.11 standard [42] (e.g., WiFi and WiFi HaLow), those encompassed as low-power wide area networks (LPWANs) (e.g., LoRa and SIGFOX), and cellular-based solutions (e.g., NB-IoT and the upcoming 5G NR releases).

Each of the aforementioned IoT wireless communication technologies has its specific use cases and advantages. However, their coexistence highlights the complex issue of interoperability. This challenge primarily derives from the utilization of distinct physical (PHY) and medium access control (MAC) layers, which impedes the seamless integration of such heterogeneous technologies into a unified ecosystem.

As IoT continues to expand its reach, achieving robust interoperability among diverse communication protocols remains a vital goal. Due to the inherent differences in IoT communication technologies at the PHY/MAC, interoperability efforts are focused on upper layers. Industry alliances, standards organizations, and consortia are working toward creating common standards and frameworks that enable devices using these protocols to communicate seamlessly with one another. For instance, the Open Connectivity Foundation (OCF) [43] is striving to establish cross-protocol compatibility.

In addition to the communication technologies described above, which allow the link between the IoT devices and the edge (or even the fog/cloud) layer, multiple communication protocols for the application layer are currently available in the IoT–edge–cloud continuum, ranging from the traditional IoT protocols (i.e., CoAP, MQTT, HTTP, AMQP, DDS, and XMPP) [44] to those expected to play an important role in the future, like gRPC [45].

Indeed, the most common way to push updates from IoT devices is through MQTT, which is a low-footprint topic-based broadcast messaging protocol. Clients connect to a broker that forwards messages to the subscribers of their respective topics. However, the semantics of the topics and the content of messages are not standardized. Most implementations use string-formatted content such as headerless CSV or JSON, but some prefer sending binary data, for example, using Protobuf serialization. MQTT can be used directly over TCP or via WebSockets. Other solutions opt for using HTTP REST to push updates directly, bypassing the need for an intermediate broker. However, HTTP REST is generally less efficient for high-frequency updates than MQTT.

The interoperability challenge extends beyond the IoT–edge–cloud continuum, encompassing the broader spectrum of data exchange and collaboration across diverse ecosystems. Standardization efforts, such as the development of common ontologies like the Smart Appliances REFerence (SAREF) [46] or the utilization of modeling tools like the Eclipse Modeling Framework (EMF) [47], play a crucial role in addressing this challenge. COGNIFOG encourages the adoption of well-known standards with the goal of facilitating the communication among heterogeneous components. An example of such an effort can be observed in the Kentyou DataHub module, where EMF is used to generate its data model in a standardized way, promoting interoperability within the ecosystem.

### 2.6. Security and Privacy

Edge computing brings into the picture the notion of decentralized intelligence, as insight generation and knowledge output are increasingly moving to the edge, close to the point where data are generated and intelligence needs to be consumed. At the same time, the highly distributed nature of the IoT–edge–cloud continuum extends the attack surface and presents a number of challenges and threats related to the stack features.

A modular and adaptable framework is essential for integrating multiple, loosely coupled tools across this spectrum. However, this integration poses several risks, including vulnerabilities and compatibility issues arising from various security protocols, authentication mechanisms, and configurations among different tools and services. Inconsistencies in integration could accidentally facilitate security breaches or lapses. Furthermore, the ubiquity of generic APIs designed for interoperability might cause potential attack vectors if not adequately secured. An unguarded API, devoid of rate limiting, could be overwhelmed in a distributed denial of service (DDoS) attack, severely impairing system functionality.

In addition, the process of transmitting and securely storing data across various nodes presents its own set of challenges, requiring rigorous encryption and secure storage protocols. The complexity of securely updating a multitude of devices, particularly within an IoT framework, emphasizes the vulnerability to security breaches. Reliance on third-party solutions introduces another layer of risk, as compromises within these external entities could have cascading effects throughout the entire framework.

The orchestration of multi-cluster systems, especially within dynamic contexts, also introduces security vulnerabilities. For instance, workloads transitioned to new clusters may not be subject to the same security policies as in their original environments, posing a risk to system integrity. Furthermore, the need to rapidly scale services during demand spikes may lead to the bypassing of certain security protocols, compromising system security for the sake of maintaining service availability.

Monitoring and analytical capabilities within distributed frameworks can also reveal vulnerabilities to AI and ML manipulations, whereby models could be tainted or misled, resulting in inaccurate predictions or analyses. On the other hand, the need for real-time monitoring can precipitate false positives or the neglect of genuine threats, as rapidly evolving data patterns may be misinterpreted as malicious activities rather than legitimate variations in user engagement. The decentralization of observability further complicates the security landscape, as isolated reports of minor anomalies from edge devices might be overlooked components of a broader, coordinated attack strategy.

The issue of interoperability and data privacy also emerges as a significant concern within this continuum. Striking a balance between system interoperability and security maintenance can be challenging, as some interoperability frameworks might bypass security checks for ease of use. An IoT device employing an outdated communication protocol may be permitted to transmit data without modern encryption standards, risking data breaches. Moreover, the transmission of data across different jurisdictions can result in regulatory non-compliance, with IoT devices in one region potentially violating privacy regulations when transmitting user data to servers in another, as exemplified by the General Data Protection Regulation (GDPR) within the European Union (EU).

## 3. A Framework for the IoT–Edge–Cloud Continuum

Encompassed within a three-year EU-funded research program and currently under development, the COGNIFOG framework embraces the cognitive fog concept to enable the IoT–edge–cloud continuum. This means that the distributed computing resources in the IoT–edge–cloud continuum will be equipped with AI-based functionalities like learning, reasoning, and decision making, allowing them to process data more intelligently and autonomously. These cognitive capabilities will significantly enhance monitoring and orchestration processes within the continuum, improving resource allocation, data transmission, and the detection of malicious activity [48].

At the IoT side, the cognitive fog will provide interoperability facilities so that IoT devices could be easily connected to the continuum and communicate with other edge-side components. At the edge side, it will operate on microservers, extending capabilities typically centralized in the cloud. On the cloud side, it will help to orchestrate all necessary resources, so that an end-to-end service will be provided in a safe and secure way in both directions. Thus, the cognitive-fog concept is multidimensional, aiming to incorporate fully decoupled building blocks that can be interconnected to suit and adapt to specific application requirements.

**Figure 1:**
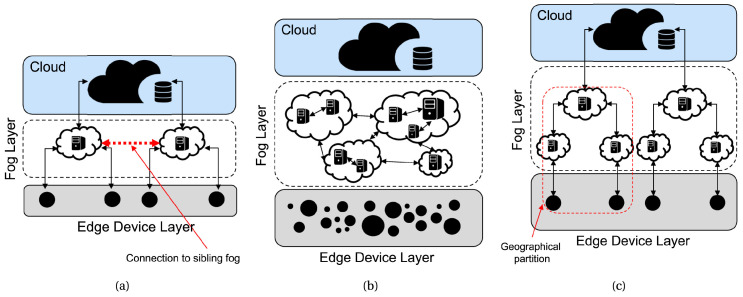
Examples of edge–fog–cloud architectures: (**a**) three-layer model; (**b**) clustered model; (**c**) tree-based model (from [23]).

According to the edge–fog–cloud architecture classification proposed in [23], whose three main models are illustrated in Figure 1, COGNIFOG adopts a clustered model, wherein fog nodes are positioned based on geographical constraints to enhance both interfog and intrafog communication capabilities. Furthermore, the COGNIFOG framework will empower the IoT–edge–cloud continuum by harnessing the synergy resulting from combining two key concepts:

- **Fog computing robustness:** By embracing the fog computing model, COGNIFOG will enable data processing closer to the data source, thus supporting reduced latency, enhanced efficiency, and optimized resource utilization. This decentralization will facilitate IoT–edge–cloud operations, promoting scalability and adaptability.
- **Cognitive orchestration:** The cognitive capabilities of COGNIFOG will emphasize the system’s capacity to autonomously process, analyze, and react to generated data. This will be empowered by advanced AI-driven analytics that provide real-time insights, proactive issue solving, and an easier understanding of complex data landscapes.

**Figure 2:**
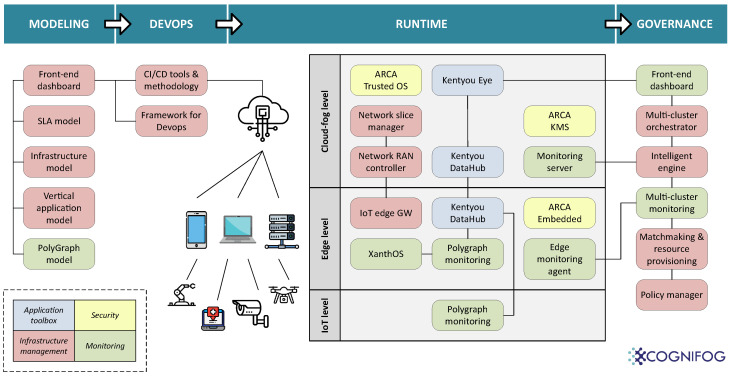
The COGNIFOG high-level architecture.

These key concepts will be made accessible to end users through an open, modular, adaptable, and secure platform that will cover the entire lifecycle of a software workload—from design and development to execution and data generation, as it can be observed in Figure 2. This architectural approach is inspired by the LF Edge Foundation [49], which brings together industry leaders to establish an open, interoperable framework for edge computing independent of hardware, silicon, cloud, or operating system (OS).

When applied to the IoT–edge–cloud continuum, this approach may offer several advantages. Firstly, it ensures interoperability, which is a fundamental aspect for integrating heterogeneous systems and technologies. Secondly, it enables the provision of distinct, loosely coupled tools, each addressing specific functionalities vital to the continuum. Thirdly, it is flexible enough to apply different configurations to its building blocks, customizing them to meet the unique demands of various use cases. Lastly, this level of flexibility also ensures the system’s resilience and longevity in a rapidly evolving technological landscape.

To put these principles into practice within the COGNIFOG architecture, the following strategies are steering the ongoing implementation:

- **Modular architecture:** The architecture is broken down into independent building blocks, so that if there is a need to update or replace a particular module, it can take place without disrupting the whole system.
- **Adoption of open standards:** Leveraging open standards ensures an easy integration of current technologies and smooth adoption of future innovations. Furthermore, this ensures interoperability, which is crucial for the seamless interaction of diverse systems within the IoT–edge–cloud continuum.
- **Flexible integration points:** By providing well-defined API endpoints and communication protocols, different tools can be integrated in various configurations. This ensures that the architecture can be tailored to heterogeneous use cases, offering a level of customization that is crucial in addressing unique challenges or requirements.
- **Continuous feedback loop:** User feedback and system performance metrics are continuously collected to refine and optimize the overall architecture.
- **Collaborative development:** Given the inspiration from the LF Edge Foundation and its emphasis on community-driven development, COGNIFOG encourages collaboration amongst its partners and the broader community. By supporting a culture of shared development, the framework can benefit from a diverse pool of expertise, ensuring robustness and innovation.

All in all, a typical user of the COGNIFOG framework should be able to use its different services to design, prepare, execute, and monitor its workload in the most secure, agile, and efficient way. The constituent building blocks of the proposed architecture have been gathered and classified in four vertical layers: the modeling layer, the DevOps layer, the runtime layer, and the governance layer. A high-level description of each layer and their building blocks is provided below.

### 3.1. The Modeling Layer

It provides end users with a set of tools to abstractly define and design the IoT–edge–cloud compute continuum before it is actually built. Nowadays, this step is highly advisable given the complexity of these new systems. A modeling layer allows end users to optimize their systems more effectively, reduce costs—especially during integration—and improve energy efficiency while also assessing future update possibilities. These tools are dedicated to describing the topology of the continuum, encompassing both hardware and software requirements. They also define service level agreement (SLA) models, application data flow models, and infrastructure models.

- **PolyGraph model:** The PolyGraph model offers a framework for analyzing systems to determine their consistency (i.e., the absence of contradictions or logical errors) and liveliness (i.e., the ability to produce a desired or acceptable outcome under a given set of initial conditions and governing rules) [39]. Model checking can then be applied to verify properties like the absence of data sample loss and/or communication starvation. It employs a formal, high-level, domain agnostic language and a deterministic MoCC data flow to design comprehensive systems made up of components (i.e., software nodes) and connectors.
- **Application and infrastructure model:** Modeling of applications and infrastructure allows for achieving a seamless implementation and operation of software systems. Whereas application modeling entails the comprehensive description of software components, services, and their interactions, infrastructure modeling encompasses the representation of the underlying computational resources: nodes, storage, networking components, and their characteristics. This information is entered by system architects in the manifest files and also in some specific entries in the COGNIFOG dashboard. Then, monitoring components also generate modeling information at runtime based on telemetry data collected from the continuum.

### 3.2. The DevOps Layer

A comprehensive continuous integration/continuous delivery (CI/CD) framework allows for automating and streamlining the software development and deployment process. Well-known open-source tools are used for better cost-efficiency, interoperability, transparency, and flexibility:

- **GitHub** for source control and tracking, code repository, code versioning.
- **Jenkins 2.470** for automated building, testing, and deployment.
- **Docker 27.0** and **Docker Compose 2.26** for bundling the developed services and components into containers using *de facto* standards.
- **SonarQube 10.6** for performing static analysis of code to detect bugs, code smells, and security vulnerabilities.
- **Kubernetes 1.30** for orchestrating the deployed services.
- **Harbor 2.7** container registry for managing, storing, and distributing the produced Docker images.
- **Portainer 2.20** for providing logging and health monitoring information for the developed and deployed applications, offering easy Docker container management through a GUI.
- **Keycloak 21.0** for handling access and identity management of the users.
- **Microsoft Teams 2024/Mail** for receiving notifications of the whole CI/CD process.
- **Ansible 2.17** for automating the deployment of the CI/CD stack.

The CI/CD platform allows users to connect and utilize tools by signing in through their GitHub account via Keycloak, serving as the single sign-on (SSO) method required for triggering deployment workflows. These workflows, initiated after the developer logs in, vary based on the chosen approach and any code-sharing security restrictions imposed by technical teams.

- In the **GitHub approach**, the process starts with a developer committing code to GitHub. This action triggers Jenkins automation via webhook, which is followed by SonarQube code analysis. If successful, an image is created and uploaded to the Harbor registry. Finally, deployment on the development server is carried out using Jenkins and Kubernetes.
- Alternatively, the **Harbor approach** begins with the developer pushing a Docker image to the Harbor registry, activating Jenkins automation, and deploying it on the development server using Jenkins and Kubernetes via webhook.

### 3.3. The Runtime Layer

The runtime layer includes the actual environments where applications operate, whether on-premises, in the cloud, on edge devices, or anywhere within the fog. It therefore encompasses servers, OSs, containers, serverless environments, databases, and other essential components necessary to host and run the applications.

#### 3.3.1. Cloud-Fog Level

- **Network Slice Manager [50]:** The Slice Manager is tasked with managing shared network infrastructure resources for the creation of *slices*. It manages a variety of resources, including compute, network, and access network resources, ensuring they are registered, retrieved, and deleted as needed. To optimize the process of resource allocation, resources are subdivided into smaller segments, known as *chunks*, which are tailored based on tenant requirements and the need for isolated networking. Moreover, the Slice Manager is responsible for overseeing the entire lifecycle of slices, which are collections of resource chunks belonging to a specific user or vertical. By effectively managing shared resources and slices throughout their lifecycle, the Slice Manager helps ensure that users and verticals have the resources they need to succeed.
- **Network RAN controller [51]:** The RAN controller is a virtualized architecture that manages and controls radio access in cellular and wireless networks. It separates the processing and control functions from the radio network and centralizes them in a data center. Consequently, it allows for greater efficiency in the network by managing and optimizing the use of radio resources, including frequency and power, based on real-time demand.
- **Kentyou DataHub:** The Kentyou DataHub is a modular platform built on top of Eclipse sensiNact [52], which is an open-source platform able to collect, analyze, and make sense of data from heterogeneous sources. The DataHub provides interoperability by means of various data bridges (also known as *southbound providers*) that facilitate access to data sources and application bridges (also known as *northbound providers*) that provide the protocols to interact with the data models held by sensiNact. The platform is constructed over OSGi, thus allowing a quick integration of heterogeneous infrastructures and the dynamic addition, update, and removal of modules at runtime. Lastly, the adaptability of this module extends beyond the cloud–fog level, as it can also be effectively deployed at the edge level.
- **Kentyou Eye: [53]** The Kentyou Eye 1.0 is a software suite that can aggregate data from multiple Eclipse sensiNact and/or Kentyou DataHub instances and provide visualization of their values. It consists of two main components:
  – **Kentyou UI:** A browser-based tool for visualizing data in various temporal and spatial granularities.
  – **Kentyou AI:** A component which comes along with a number of integrated AI/ML services allowing forecasts, optimizations, and AI-based decision making. It supports new AI services by plugging them into its AI service management mechanism, allowing parameterized executions to provide multiple results from the same AI model, the persistent storage of models/results, and dynamic re-training.
- **ARCA Trusted OS [54]:** A hardened Linux-based micro-distribution, which provides robust protection against system intrusions and prevents data compromise within containers deployed on-premises, on the cloud, and at the edge. At its core, ARCA Trusted OS combines the strength of a hardened OS with a secure Kubernetes orchestrator, ensuring the security and isolation of the infrastructure’s critical components, and minimizing the risk of unauthorized access or tampering. By leveraging confidential computing technologies and hardware-based security features, ARCA Trusted OS enables applications and workloads to operate in secure enclaves, ensuring that sensitive data remain protected even from the underlying infrastructure.
- **ARCA Key Management System (KMS) [55]:** ARCA KMS provides access to cryptographic capabilities using different types of cryptographic backends like OpenSSL, HSMs, TPMs, and post-quantum crypto cores. The used backend is completely transparent to the user application who only interacts with the gRPC API. The RPC requests are transported over HTTP/2 and can be encoded in various ways, including protocol buffers.
- **Monitoring server:** Based on state-of-the-art technologies focused on decentralized computing observability [56], the monitoring server operates as a multi-instance application strategically replicated across various cloud-level nodes to ensure high availability for data exporters (i.e., *agents*), transmitting telemetry data. These cloud-level nodes are responsible for the collection and aggregation of telemetry data, including both static and dynamic performance metrics, as well as energy consumption data from downstream nodes. Once gathered, the data are forwarded to a multi-cluster monitoring controller, which processes it, manages its long-term storage, and presents it to the governance layer components of the COGNIFOG framework, thereby supporting AI-powered optimized system operations.

#### 3.3.2. Edge Level

- **IoT edge gateway (GW):** Based on a modular multi-interface physical platform, this module is designed to facilitate heterogeneous networking in the IoT–edge–cloud continuum. It acts as an intermediate element connecting low-capable devices at the far edge with the rest of the fog/cloud infrastructure. Beyond providing flexible and transparent interactions among diverse devices and technologies, the IoT edge GW can also offer additional processing capabilities, such as data filtering, managing flow priorities, and executing low-latency actions based on locally available information. This enhances scalability, overall efficiency, and responsiveness of IoT systems, enabling more effective and intelligent data handling at the edge of the network.
- **ARCA Embedded:** ARCA Embedded is the distribution of ARCA Trusted OS designed for ARM architecture. The distribution is based on the same security principle: providing a trusted execution environment (TEE) for sensitive edge workloads. Similar to ARCA Trusted OS, ARCA Embedded features a TPM-based secure boot, immutable file systems, and a seamless system updater. ARCA Embedded leverages the use of ARM Trustzone [57] whenever this hardware extension is available, providing a confidential computing environment for sensitive cryptographic operations.
- **Edge monitoring agent:** Telemetry data, encompassing static resources (e.g., processor capacity, memory availability, storage, and network capabilities), dynamic performance metrics, and the energy consumption (distinguishing between renewable, or *green*, energy sources and those associated with carbon emissions) of edge nodes, are collected by the monitoring agent and relayed to the already presented monitoring server.

#### 3.3.3. IoT Level

- **PolyGraph monitors:** During the execution of a system, some phenomena are not expected. The OS (e.g., a Linux kernel) interferes with the system by managing the priorities of the services. Those events can change the execution of applications running on top of the OS. The monitors capture the behavior of the applications and verify that everything is executing as expected. PolyGraph monitors are generated from the PolyGraph model, which will be securely embedded in far-edge devices [58]. Some monitoring actors will run using the automated OS generation framework XanthOS [59], which allows building secure, performant, and custom system software.

### 3.4. The Governance Layer

**Figure 3:**
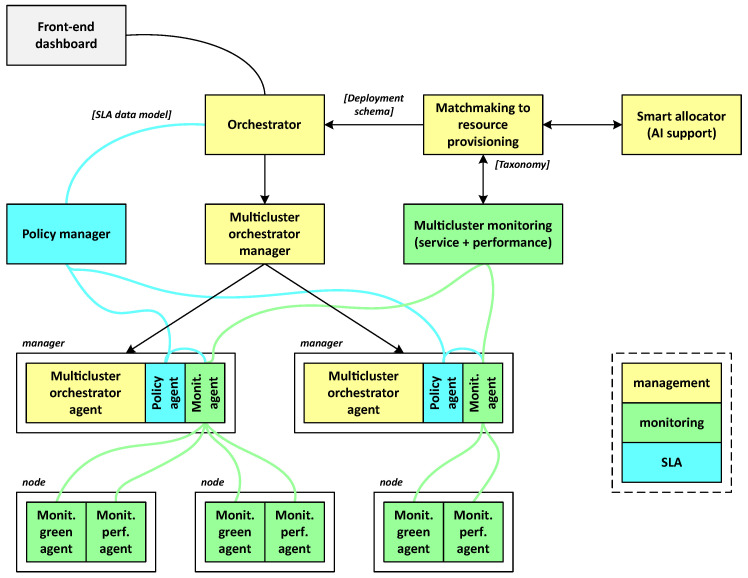
Governance layer architecture based on a multi-cluster approach (based on [60]).

This layer incorporates tools and practices for intelligent orchestration, monitoring, logging, auditing, and reporting (see Figure 3). It ensures adherence to proper processes, the enforcement of policies, software security, data protection, and facilitates the tracing and resolution of any issues.

- **Front-end dashboard:** The front-end dashboard offers an intuitive interface for users to interact with the cognitive fog network, along with APIs that facilitate the integration of third-party applications. It is designed to be user-friendly, enabling application developers to create API calls with precise control, simplifying the complexity involved in deploying advanced services. This approach enhances the compatibility between external applications and the COGNIFOG framework, fostering an environment of interoperability.
- **Multi-cluster orchestrator:** It is composed of two main parts that communicate with each other to exchange deployment information about the desired and the current status of the application workloads across the IoT–edge–cloud continuum:
  – **Multi-cluster orchestrator manager:** This service is responsible for deploying the required application resources across the clusters that have been on-boarded to the COGNIFOG framework. Information related to the application description and deployment requirements will be received from two other governance layer components: the front-end dashboard and the intelligent engine.
  – **Multi-cluster orchestrator agent:** This component runs on every cluster manager that has been on-boarded to the COGNIFOG framework. It receives the application description and deployment requirements from the multi-cluster orchestrator manager and allocates those resources across its managed cloud, fog, and edge nodes.
- **Intelligent engine:** The intelligent engine, also known as smart allocator, operates in tandem with the multi-cluster orchestrator, providing it with guidance on how to allocate application workloads across the available infrastructure. This decision-making process considers various factors, such as network latency, data proximity, and the availability of hardware resources, as well as their power consumption.
  Due to the synergies with the also ongoing Horizon Europe project CODECO [61] in terms of technical challenges and participating partners, the smart allocator conceived within the COGNIFOG framework leverages its open-source scheduling and workload migration (SWM) module [62]. SWM comprises two subcomponents: the scheduler, which handles the simultaneous placement of all pods in an application group, and the workload placement solver, which determines the optimal placement of application workloads on available nodes.
  To optimize this allocation process and enhance the overall system efficiency and performance, the smart allocator may be powered by an AI algorithm. Although fully functional AI modules are not yet available in the COGNIFOG framework, several reinforcement learning (RL) techniques are currently under study given RL’s effectiveness in solving resource allocation problems within the IoT–edge–cloud continuum [63].
  In the COGNIFOG context, RL agents would be trained using data collected from monitoring agents, leveraging insights derived from the nodes’ operational dynamics and energy utilization patterns. Once trained, the validated RL agents would be integrated into the smart allocator, which would then be able to provide RL-based placement plans to the orchestrator. When run iteratively, this process would refine the system’s configuration to achieve an optimal solution that balances multiple criteria effectively.
- **Policy manager:** The policy manager oversees the compliance of SLA policies of deployed applications before deployment and at runtime. It constantly analyzes feedback received from the monitoring server against application policies. Policy agents, distributed across clusters, implement these policies at the node level, coordinating with the central policy manager for guidance and updates. Furthermore, if integration with COGNIFOG’s intelligent systems becomes viable, the policy manager could potentially leverage predictive data to perform both reactive and proactive actions, including workload adjustments, relocations, scaling, and migrations.

## 4. Toward Cognitive Connectivity: The Case of Dynamic Bandwidth Management

One of COGNIFOG’s primary objectives is to implement essential features to enable cognitive connectivity: that is, to ensure end-to-end connectivity between services deployed across the IoT–edge–cloud continuum with self-adaptability. The interaction between COGNIFOG’s building blocks will empower the system to (1) *monitor* the current state of the connectivity layer, (2) *determine* the most efficient configuration of network resources, and (3) *apply* the necessary changes to achieve an optimal configuration dynamically.

In particular, one of the features of cognitive connectivity is to provide dynamic BW management across network links in a system that supports multiple applications with heterogeneous traffic patterns and priorities. This is especially useful, for instance, in scenarios such as emergency response operations, where network infrastructure is scarce, prone to congestion, or directly unavailable. In such cases, employing BW management techniques to delay the flow of certain types of network packets can effectively ensure network performance for higher-priority applications.

### 4.1. Proof of Concept for Bandwidth Management

To exemplify the potential of BW management, a proof of concept (PoC) was developed where network (re)configurations were performed manually. Specifically, a cluster consisting of three interconnected VMs (hereafter referred to as *nodes*) was deployed using K3s [64], a lightweight and easy-to-install container orchestration platform based on Kubernetes, and Cilium [65], a network manager tool. In this setup, one node acted as the control plane node, and the other two acted as worker nodes. One worker node (i.e., *worker #1*) simulated an edge node generating both TCP and UDP traffic addressed to another worker node (i.e., *worker #2*) located in the cloud.

**Figure 4:**
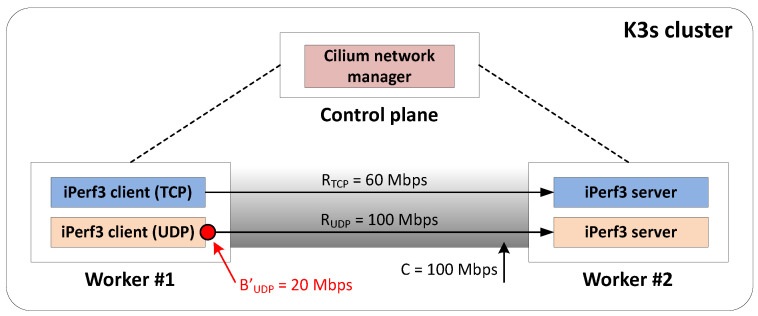
The K3s cluster designed for the PoC. The red bullet indicates the activation of the egress BW limitation only at the final stage of the performance evaluation.

Consider a use case in which all TCP traffic came from high-priority services, while UDP traffic corresponded to low-priority services. The data generation rates of applications over TCP and UDP were $R_{\mathrm{TCP}}\;=\;60$ Mbps and $R_{\mathrm{UDP}}\;=\;100$ Mbps, respectively. Additionally, the link capacity between worker nodes was artificially limited to C=100 Mbps to explicitly simulate a potential situation where the link could not support the data transmission of both TCP and UDP applications simultaneously. TCP and UDP traffic were generated using iPerf3 3.1.3, which is a traffic generator and measurement tool [66]. More concretely, two different iPerf3 client instances (i.e., *pods*) were deployed in worker #1. Similarly, worker #2 contained two different iPerf3 server pods that received the corresponding traffic (see Figure 4).

The PoC was run over a T=6 min period, divided into three distinct stages of 2 min each, with different network conditions:

- **Stage 1** (minutes 0 to 2): Only TCP high-priority traffic was present on the link.
- **Stage 2** (minutes 2 to 4): TCP high-priority and UDP low-priority traffic were present on the link simultaneously.
- **Stage 3** (minutes 4 to 6): TCP high-priority and UDP low-priority traffic were present on the link simultaneously. However, the configuration of the iPerf3 pod handling UDP traffic was modified on-the-fly from the control plane node at minute 4 to limit its BW to 20 Mbps (i.e., $B_{\mathrm{UDP}}^{\prime }\;=\;20$ Mbps).

**Figure 5:**
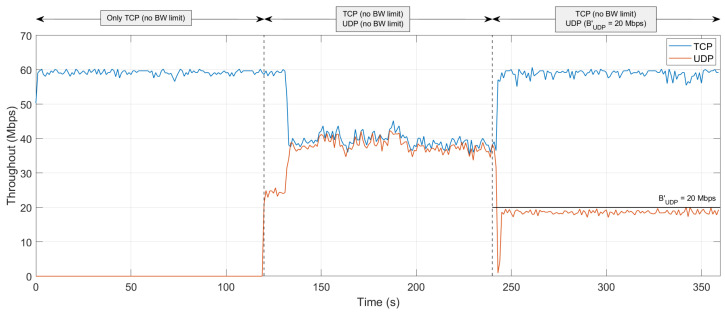
Resulting throughput of TCP high-priority and UDP low-priority traffic.

Figure 5 illustrates the resulting throughput (*S*) for each type of traffic on the observed link during the three analyzed stages. As expected, $S_{\mathrm{TCP}}\;\approx \;R_{\mathrm{TCP}}$ during Stage 1, as no other traffic is present on the link. However, when UDP traffic is introduced from minute 2 on, both types of traffic compete for the under-dimensioned capacity of the link, thus resulting in $S_{\mathrm{TCP}}\;\approx \;40$ Mbps ($S_{\mathrm{TCP}}\;<\;R_{\mathrm{TCP}}$) and $S_{\mathrm{UDP}}\;\approx \;40$ Mbps ($S_{\mathrm{UDP}}\;\ll \;R_{\mathrm{UDP}}$). Lastly, once the BW limitation of UDP traffic is activated from minute 4 on, high-priority TCP traffic effectively arrives at its destination (i.e., $S_{\mathrm{TCP}}\;\approx \;R_{\mathrm{TCP}}$) at the cost of degrading low-priority UDP traffic (i.e., $S_{\mathrm{UDP}}\;⋘\;R_{\mathrm{UDP}}$, since $S_{\mathrm{UDP}}\;\leq \;B_{\mathrm{UDP}}^{\prime }$).

### 4.2. On the Integration of the Bandwidth Management into the COGNIFOG Stack

The conducted PoC exemplifies how network management techniques can effectively combat BW starvation and contention issues caused by resource-intensive services on a shared network link. In this case, the BW limitation technique was manually implemented, but the final version of the COGNIFOG framework shall integrate it with advanced monitoring, orchestration, and AI modules to achieve true cognitive connectivity that automatically adapts services to dynamic network conditions.

**Figure 6:**
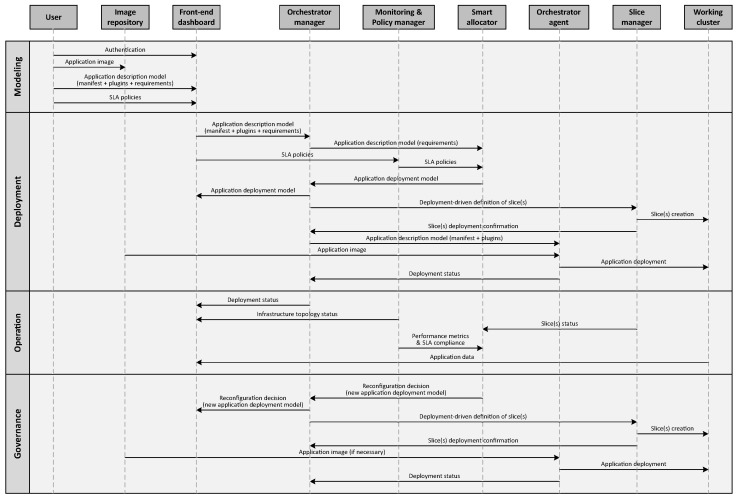
High-level interaction flow diagram of the COGNIFOG stack.

This section elaborates on how the COGNIFOG framework could integrate the BW management feature, which is just one of the multiple functionalities encompassed under the flexible and dynamic provisioning and management of computing, storage, and networking resources. To illustrate this, a high-level interaction flow diagram of the main elements of the COGNIFOG stack is provided in Figure 6.

Assuming proper deployment and interconnection of the depicted COGNIFOG elements, their interactions can be grouped into four main stages:

1. **Modeling:** After the user authentication, application image(s) are uploaded to an image repository. SLA policies and the application description model (consisting of manifest files, plugins, and specific requirements) are also defined by the user in the front-end dashboard.
2. **Deployment:** The requirements of the application description model, along with the SLA policies, are received by the smart allocator. With these inputs, it is able to design an initial application deployment model, which is then communicated to the orchestrator manager and the user via the front-end dashboard. Subsequently, slices containing computing, network, and access network chunks are created under a *deployment-driven* model on the resources of the working cluster [50]. Finally, the application is deployed on the targeted nodes according to the application description model.
3. **Operation:** During operation, the end user receives not only application data but also updated information on the status of the deployment itself. For its part, the smart allocator also receives information on the status of the slices, performance metrics, and SLA compliance, which feeds its intelligent engine and may trigger a deployment reconfiguration if necessary.
4. **Governance:** The depicted interaction flow shows how a new application deployment model is designed by the smart allocator. This process may be initiated after detecting non-compliance with SLA policies. As in the initial deployment, the model is communicated to the orchestrator manager and the user via the front-end dashboard. The slices and the application are then redeployed according to the new model to enhance overall system efficiency and performance.

Returning to the specific case of the BW management functionality, the final version of the COGNIFOG stack shall automate most of the processes described in the PoC. For instance, during the modeling stage, a user could define an SLA policy for TCP high-priority traffic to ensure a specific value of $S_{\mathrm{TCP}}$. Then, in the operation stage, if the smart allocator detects that the value of $S_{\mathrm{TCP}}$ reported by the performance metrics of the monitoring system does not meet the SLA policy, it would automatically initiate the reconfiguration of the deployment as part of the governance stage.

Based on the information of the infrastructure (i.e., available resources and status) and considering the compliance with the SLA policy, the smart allocator shall decide to limit the available BW for the UDP traffic corresponding to low-priority services without further human intervention. This could be achieved by means of simple decision-making procedures or, alternatively, through an AI module responsible for providing the optimal configuration based on constraints, requirements, and making use of past information.

## 5. COGNIFOG Evaluation in an IoT Connectivity Testbed

A preliminary implementation of a selected set of connectivity-related building blocks from the COGNIFOG framework was conducted within a controlled IoT testbed environment. This exercise aimed to validate the proper deployment and interconnection of these modules on heterogeneous infrastructure elements across the IoT–edge–cloud continuum. It then sought to demonstrate how the interplay of these modules currently facilitates the effective operation of an IoT application and how they could also contribute to cognitive connectivity capabilities in the future when integrated with the intelligent monitoring and orchestration elements from the COGNIFOG framework.

An e-Health application was simulated over this testbed environment, where a number of smartwatches worn by users monitored their activity and health status, periodically transmitting the acquired data to an external server. The COGNIFOG framework was here only responsible for the deployment and management of the different services into the available computing and networking infrastructure. Ultimately, this setup would allow for the effective visualization, storage, and treatment of the data collected by the smartwatches as well as the immediate initiation of suitable actions in case predefined alarms or emergencies were detected.

### 5.1. Architecture and Main Operation

**Figure 7:**
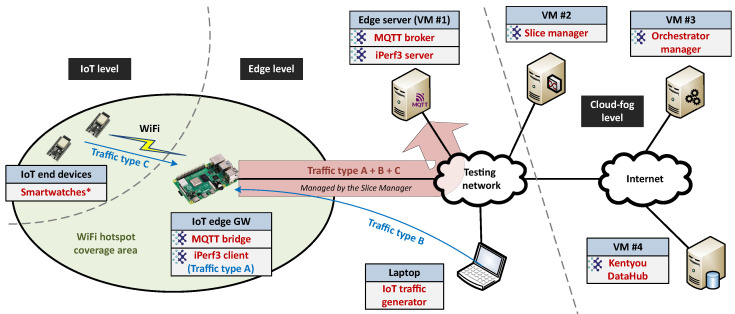
COGNIFOG testbed architecture. Containerized services than can be remotely deployed by the orchestrator manager include the COGNIFOG logo.

The set of modules from the COGNIFOG framework included in the testbed spanned across the IoT–edge–cloud continuum within a single K3s-based cluster with Cilium as the network manager. Real end devices simulating the operation of smartwatches were located at the IoT level. The acquired information from these devices (i.e., traffic type C) was transmitted wirelessly to the IoT edge GW, which was located at the edge level. This element forwarded the received data to the edge server together with two other traffic flows artificially generated: a UDP background stream (i.e., traffic type A) and synthetic information from a smartwatch simulator (i.e., traffic type B). A diagram of the proposed testbed architecture is shown in Figure 7.

At the cloud–fog level, an instance of the Slice Manager oversees the creation and management of network slices across the communication link connecting the IoT edge GW and the edge server. In the testbed setup, distinct slices were allocated for each type of traffic. Alternatively, in other potential scenarios, slices could also be utilized to segregate multi-tenant workloads within the same cluster. Other elements of the cloud–fog level are the Kentyou DataHub (responsible for retrieving, storing, and facilitating the visualization of information stored at the edge server) and the orchestrator manager (which coordinates the remote deployment and management of these elements across the infrastructure subscribed to the COGNIFOG framework by means of the corresponding orchestration agents).

Any new module aiming to be integrated into the COGNIFOG framework should meet three basic requirements: (1) it should be IP reachable, (2) it should use K3s, and (3) it should run the COGNIFOG orchestration agent. Once these conditions are satisfied, when the orchestrator manager executes a resource discovery process, it would *see* a new element that could be incorporated into a managed cluster and ultimately oversee the deployment and management of its services.

### 5.2. Implementation

#### 5.2.1. IoT Level

Espressif ESP32-based development boards [67], equipped with a dual-core MCU and a WiFi interface, have been utilized at the IoT level as far-edge devices. ESP32 offers abundant I/O options for interfacing with various devices (e.g., sensors and actuators), low power consumption, and advanced security features (e.g., secure boot). These boards simulate the typical operation of smartwatches connected to the network via WiFi technology and using TCP/IP. Synthetic data were encapsulated in MQTT messages, which were formatted according to the JSON specification defined by CloudEvents [68].

The firmware employed on top of the ESP32 boards was Tasmota [69], which was an open-source firmware designed for home automation and smart devices that allows users to develop their own IoT applications with higher flexibility and control. In terms of upper communication protocols, it supports MQTT, HTTP, and Matter, among others.

Lastly, apart from a couple of real IoT devices, a simulator of smartwatches was also implemented and integrated into the evaluation testbed. Executed from a laptop, it allowed the creation of multiple concurrent, independent instances of simulated smartwatches that sent synthetic data periodically through MQTT according to the CloudEvents specification.

#### 5.2.2. Edge Level

The hardware running the IoT edge GW is a Raspberry Pi 4 Model B [70]. It is a versatile and affordable single-board computer, widely employed in edge and fog computing paradigms [71], featuring a powerful quad-core processor, up to 8 GB of RAM, USB ports, Gigabit Ethernet, WiFi, and Bluetooth. Two different OSs have been validated on this platform for the testing campaign: Debian GNU/Linux 12 and ARCA Trusted OS for ARM architectures.

The Raspberry Pi has been configured to use its Ethernet connection for reliable uplink communication with the edge server while also acting as a WiFi hotspot at 2.4 GHz to provide versatile wireless connectivity for IoT end devices. An instance of K3s was deployed with two running services: an MQTT bridge and an iPerf3 client.

The edge server consisted of a VM located in the same premises as the IoT end devices and the IoT edge GW. Similarly to the IoT edge GW, the edge server also contained an instance of K3s with two services: an MQTT broker and an iPerf3 server. Ideally, the final e-Health application would also perform local processing on the edge server of contextual information coming from multiple IoT edge GWs.

#### 5.2.3. Cloud–Fog Level

The cloud–fog level of the testbed environment consisted of three VMs running over K3s: a containerized version of the Slice Manager, the orchestrator manager, and the Kentyou DataHub, respectively. It is worth noting here that all three VMs were placed in different and distant locations to demonstrate the support for decentralized architectures of the COGNIFOG framework.

### 5.3. Network Traffic Types and Characteristics

In the presented IoT testbed environment, the link between the IoT edge GW and the edge server may accommodate three distinct traffic flows. The characteristics of each traffic type are described below:

- **A. UDP background streaming flow:** A continuous flow of UDP traffic with a constant data generation rate ($R_{\mathrm{UDP}}$) was used to assess the network’s performance under streaming-like traffic loads, such as those generated by a CCTV surveillance camera. To generate this traffic flow, a containerized version of the iPerf3 tool was deployed to both the IoT edge GW (acting as the iPerf3 client) and the edge server (acting as the iPerf3 server).
- **B. Smartwatch simulator:** A number of $n_{\mathrm{SW}}$ smartwatches were simulated from a web application run in a laptop belonging to the testing network. Every simulated smartwatch sent five messages ($k_{\mathrm{SW}}\;=\;5$) via Ethernet to the MQTT bridge (with QoS level equal to 0) located at the IoT edge GW every $t_{\mathrm{SW}}$ seconds with synthetic information about vital signs (namely, heart rate and oximetry), steps, and other complementary information (namely, location and battery level). The MQTT QoS level was set to 0, ensuring best-effort delivery with no acknowledgment by the receiver and, consequently, providing the same guarantee as the underlying TCP protocol. The IoT edge GW, in turn, forwarded those messages to the MQTT broker located at the edge server. The payload size of each of these messages was roughly 350 bytes ($l_{\mathrm{SW}}\;=\;350$ B).
  The simulated version of the smartwatches employed in this study greatly simplifies the communication challenges associated with a shared channel, such as those potentially encountered in a real-world WiFi-based scenario. Although the mitigation of such issues is out of the scope of the present paper, it is important to note that a key component of the future fully integrated COGNIFOG framework, the *network RAN controller*, has been specifically designed to manage radio access in cellular and wireless networks.
- **C. Real IoT devices:** Two ESP32 boards ($n_{\mathrm{ESP}}\;=\;2$) were integrated into the testbed, sending one message ($k_{\mathrm{ESP}}\;=\;1$) via WiFi to the MQTT bridge (with QoS level equal to 0) located at the IoT edge GW every $t_{\mathrm{ESP}}$ seconds with synthetic information about the heart rate. As in the previous case, the IoT edge GW also forwarded those messages to the edge server. The payload size of these messages was roughly 250 bytes ($l_{\mathrm{ESP}}\;=\;250$ B).

### 5.4. Test Description and Results

The effectiveness of the implemented COGNIFOG framework in running the e-Health application was evaluated through a comprehensive test. The primary focus of this evaluation was on addressing connectivity issues to ensure the accurate, timely, and seamless transmission of data collected by IoT devices even when coexisting with other types of traffic sharing the same network resources.

Specifically, the impact of a UDP background streaming flow (i.e., traffic type A) on the continuous delivery of MQTT messages from both the simulated e-Health application (i.e., traffic type B) and the real IoT devices (i.e., traffic type C) was quantified. Furthermore, the test incorporated the capability to limit the BW for specific services, thereby ensuring available network resources for other higher-priority services, as presented in Section 4.

Performance results were obtained for the link connecting the IoT edge GW and the edge server, where all outgoing traffic from the IoT edge GW shared the capacity of a single Ethernet-based uplink connection, which was denoted as $C_{\mathrm{UL}}$. (The wireless link between the two IoT devices and the IoT edge GW is assumed to remain stable for the entire test duration, not introducing any major variations in the results obtained.) By default, this value amounts to 1 Gbps within the premises of the testbed environment. However, $C_{\mathrm{UL}}$ was deliberately constrained to 4 Mbps using Wondershaper—a command-line utility for limiting an adapter’s capacity [72]. This limitation was intended to emulate more realistic operational conditions of IoT-based e-Health applications, where the system might be subject to lower communication technology capacities, reduced BW allocation, or network saturation during emergencies [73]. No further restrictions were applied in the downlink.

To better illustrate the impact of several traffic flows sharing the same link, a UDP background streaming flow was explicitly designed to exceed the network’s capacity. This scenario intends to replicate situations such as an unexpected degradation of the uplink communication link or the sudden activation of one or multiple traffic sources, like those generated by alarm-triggered surveillance cameras. In this case, the UDP data generation rate ($R_{\mathrm{UDP}}$) was set to 8 Mbps.

The entire test lasted 10 min (T=10 min; from 12:00:00 to 12:10:00) with the configuration parameters detailed in Table 1. During this time, a continuous stream of MQTT messages was first transmitted from both simulated and real IoT devices to the IoT edge GW and then forwarded to the edge server, adhering to the characteristics of traffic types B and C, respectively. *T* was divided into 3 differentiated stages (where $T\;=\;T_{1}\;+\;T_{2}\;+\;T_{3}$) with the following characteristics:

- **Stage 1** ($T_{1}\;=\;2$ min; from 12:00:00 to 12:02:00): Only MQTT traffic from types B and C was present on the link between the IoT edge GW and the edge server.
- **Stage 2** ($T_{2}\;=\;4$ min; from 12:02:00 to 12:06:00): Traffic from types A, B, and C was present on the same link simultaneously.
- **Stage 3** ($T_{3}\;=\;4$ min; from 12:06:00 to 12:10:00): Traffic from types A, B, and C was present on the same link simultaneously. However, the configuration of the iPerf3 pod handling UDP traffic was modified on the fly by the Slice Manager at the beginning of this stage to limit its egress BW to 2 Mbps (i.e., $B_{\mathrm{UDP}}^{\prime }\;=\;2$ Mbps).

The conducted assessment involved analyzing only the messages from traffic type C: that is, those retrieved from the MQTT broker that were originally sent by the two ESP32 boards acting as smartwatches. These messages also contained timestamps, marking the exact moment each message was generated. As a consequence, some related performance metrics were computed, such as the end-to-end latency (*L*), the delay between consecutive messages (*D*), the jitter (*J*), and the packet–delivery ratio (PDR).

**Figure 8:**
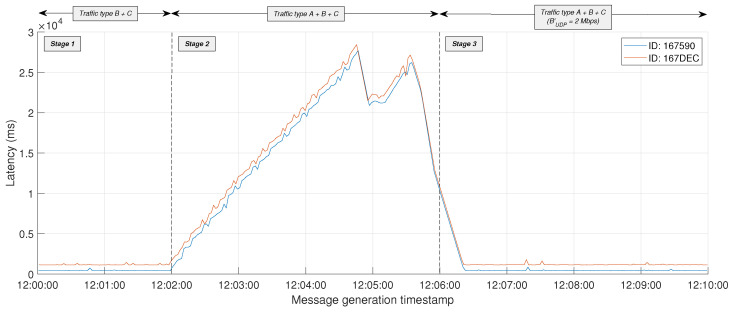
End-to-end computed *L* of the MQTT messages sent by the two IoT devices.

As shown in Figure 8, during Stage 1, with no background traffic, the *L* of MQTT messages sent by the two analyzed IoT devices (IDs #167590 and #167DEC) remains stable with values around 1 s. However, as soon as UDP background traffic is introduced in Stage 2, *L* starts to grow until reaching even more than 25 s in both evaluated devices between 12:04:00 and 12:05:00.

*L* from both IoT devices also experiences remarkable drops during Stage 2. These are caused by the queue and buffer management mechanisms operating in network devices, which, when facing saturation, discard packets to manage the existing congestion. Be that as it may, a few seconds after the limitation of BW to background traffic is applied at the beginning of Stage 3, *L* values smoothly return to the previous stable ranges from Stage 1.

**Figure 9:**
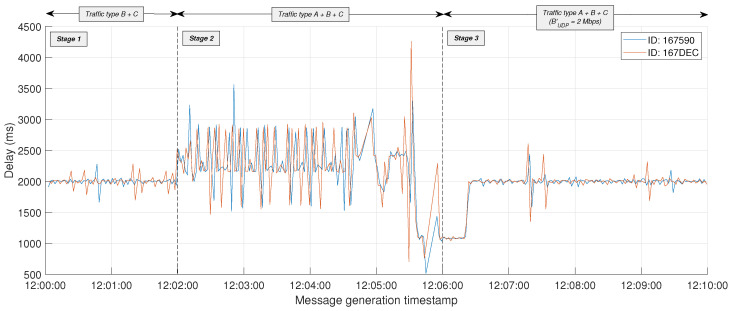
Computed *D* of the MQTT messages sent by the two IoT devices.

*D* is considered here as the time difference between two consecutive received MQTT messages. As depicted in Figure 9, *D* remains relatively stable in Stage 1, with $D\;\approx \;t_{\mathrm{ESP}}$ and $t_{\mathrm{ESP}}\;=\;2$ s. However, during most of Stage 2, $D\;>\;t_{\mathrm{ESP}}$, occasionally reaching peaks over 3 s, which was primarily due to the existing background traffic that disrupts the regular reception of MQTT messages. In fact, the internal dynamics of queue and buffer management mechanisms under congested conditions contribute significantly to the fluctuations observed in *D* during this stage. But again, the benefits of applying the BW limitation become evident in Stage 3, where $D\;\approx \;t_{\mathrm{ESP}}$ after a brief transient period of approximately 30 s.

**Figure 10:**
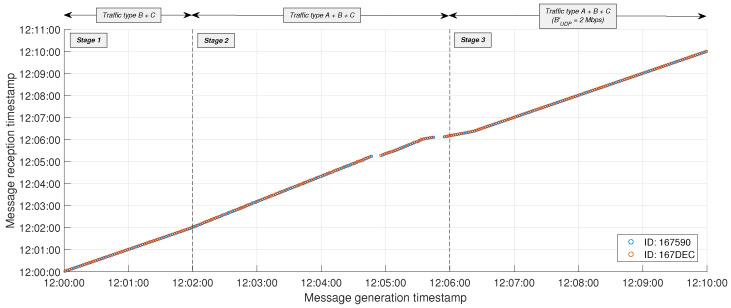
Timestamp comparison between the MQTT message generation in the IoT devices and the corresponding reception at the edge server.

Figure 10 represents with a bullet each MQTT packet from traffic type C correctly received by the edge server. Bullets are referenced by the message generation timestamp on the X-axis and the reception timestamp on the Y-axis. Consequently, the slope of the line formed by the succession of bullets is related to the timely transmission of periodic MQTT packets, while any gaps indicate packet loss. As it can be observed, the stability of the system during Stage 1 is disrupted in Stage 2, where the increasing degradation in the continuous reception of packets results in two noticeable time periods in which all MQTT packets are lost (just before 12:05:00 and 12:06:00). Nevertheless, the BW limitation implemented in Stage 3 facilitates the rapid transmission of stored MQTT packets at the beginning of this stage, subsequently restoring the stability observed in Stage 1.

All metrics of interest from IoT devices #167590 and #167DEC have been compiled in Table 2. Overall, the BW limitation for UDP traffic in Stage 3 has allowed MQTT traffic from real IoT devices to achieve performance in terms of latency, delay, and jitter similar to that observed during Stage 1 when no UDP traffic was present on the link. The reliability of MQTT packets also benefits from the BW limitation of UDP traffic, as the PDR for both IoT devices increases from 89.6% and 88.8% in Stage 2 to 100% in Stage 3.

Lastly, and as expected, the performance of UDP-based background traffic (with $R_{\mathrm{UDP}}\;=\;8$ Mbps) was significantly degraded following the application of the BW limitation on the communication link between the IoT edge gateway and the edge server. Specifically, from the analysis of the obtained iPerf3 logs, the computed throughput for this type of traffic ($S_{\mathrm{UDP}}$) decreased from $S_{\mathrm{UDP}}\;=\;2.79$ Mbps in Stage 2 (when no BW limitation was applied) to $S_{\mathrm{UDP}}^{\prime }\;=\;1.95$ Mbps in Stage 3 (when $B_{\mathrm{UDP}}^{\prime }\;=\;2$ Mbps).

All in all, the tests conducted in the IoT testbed demonstrate how the BW management feature effectively enhances connectivity performance by ensuring the proper operation of high-priority services over low-priority ones on a shared network link. Although the feature was manually activated in these tests, it stands to benefit from the governance building blocks of the COGNIFOG framework in the future, potentially operating in an automated and intelligent manner.

In fact, the same metrics used here to evaluate connectivity performance (i.e., latency, delay, and PDR) could be employed to assess whether the SLA policies defined by a potential COGNIFOG user are being met. As detailed in Section 4.2, the monitoring and the policy managers within the COGNIFOG framework would be responsible for collecting data on performance metrics and SLA compliance, respectively. These data would then be delivered to the smart allocator, which, considering the status of infrastructure resources, would initiate a reconfiguration of service deployment if necessary. This could include, as demonstrated in this testbed, the activation of BW limitations for certain services.

## 6. Conclusions and Future Work

As modern systems are increasingly integrating sensors and actuators with built-in network connectivity, they are adopting architectures that distribute computing workloads to remote resources. The subsequent emergence of new paradigms such as the IoT and edge/fog computing motivates the implementation of advanced orchestration of these decentralized computing resources across heterogeneous environments, thus blending IoT, edge, fog, and cloud computing into a cohesive computing continuum [74].

The COGNIFOG framework aligns with this vision by integrating advanced cognitive functions into fog computing architectures. It aims to ensure the efficient operation of next-generation applications that require autonomous data processing and robust computing capabilities along the IoT–edge–cloud continuum. This paper presents the architecture and the key building blocks of the COGNIFOG framework, which is still under development as part of the three-year EU-funded research project in which it is embedded. Special emphasis is placed on the connectivity modules, which, due to their advanced level of development, have enabled the design of a PoC that validates the core functionality of BW management and paves the way for the concept of cognitive connectivity.

A pilot version of the COGNIFOG framework has been implemented and evaluated in the context of an e-Health application, where it handles data acquisition from IoT devices and manages processing tasks across heterogeneous computing resources. Although this preliminary version only includes some building blocks, it is capable of not only deploying and managing all the necessary services for a typical IoT application across the available resources of the IoT–edge–cloud continuum but also providing improved performance in terms of connectivity, even in situations of network congestion or instability.

Looking ahead, the short-term future work within COGNIFOG entails the integration of the remaining building blocks, significantly boosting the framework with advanced capabilities for real-time adaptability and security. Moreover, the framework will undergo further evaluations not only to verify its effectiveness in the proposed e-Health application with fully functional smartwatches but also to extend its applicability to two additional use cases in the areas of Smart Cities and Industry 4.0, broadening its impact and utility in dynamic real-world environments.

But beyond the scope of the COGNIFOG framework, further challenges persist in the domain of the IoT–edge–cloud continuum. For instance, extending the presented orchestration/management capabilities to highly constrained hardware platforms is a significant challenge. The existing heterogeneity in both hardware and operating software of these platforms, very common in IoT end devices, cannot be managed using conventional architectures.

In this context, some potential solutions involve the use of firmware that can be selected and deployed on the fly, the allocation of purpose-designed firmware functions in isolated memory blocks, or the implementation of protocols to modify specific configuration parameters. However, more ambitious approaches in the current literature suggest using meta-OS reference architectures, which would facilitate creating a real peer-to-peer continuum for IoT applications, thereby addressing the limitations of conventional models [22].

Such architectures may help overcome issues resulting from the limited computing power and energy capacity of IoT and edge devices, which typically restrict their operations to their designated layers [75]. In contrast, swarm intelligence—defined as the ability of a swarm (or multiple agents) to handle complex tasks unachievable by individual entities without a central coordinator—promotes a shift from programming individual devices to fostering dynamic and cooperative groups of nodes. This shift would enable distributed and self-organized collective intelligence, leading to an optimal utilization of the available computing power across diverse cognitive devices at the edge [76].

## Figures and Tables

**Table 1:** Test configuration parameters.

		**Parameter**	**Description**	**Value**
		*T*	Test duration	600 s
		$C_{\mathrm{UL}}$	UL capacity	4 Mbps
**Traffic** **type**	**A**	$R_{\mathrm{UDP}}$	UDP traffic generation rate	8 Mbps
		$B_{\mathrm{UDP}}^{\prime }$	UDP bandwidth limitation	2 Mbps
	**B**	$n_{\mathrm{SW}}$	Number of simulated smartwatches	150
		$k_{\mathrm{SW}}$	Number of messages per smartwatch and transmission period	5
		$t_{\mathrm{SW}}$	Transmission period of smartwatches	2 s
		$L_{\mathrm{SW}}$	Length of a smartwatch message	350 B
	**C**	$n_{\mathrm{ESP}}$	Number of real IoT devices	2
		$k_{\mathrm{ESP}}$	Number of messages per IoT device and transmission period	1
		$t_{\mathrm{ESP}}$	Transmission period of IoT devices	2 s
		$L_{\mathrm{ESP}}$	Length of an IoT device message	250 B

**Table 2:** Metrics of interest of the performed test for the two analyzed IoT devices. (* Statistics from Stage 3 refer to its non-transient period: that is, from 12:06:30 to 12:10:00.)

Metric		ID #167590			ID #167DEC		
		Stage 1	Stage 2	Stage 3 *	Stage 1	Stage 2	Stage 3 *
**L (ms)**	$\bar{L}$	452.78	16,192.96	438.87	1167.00	17,061.18	1165.20
	$L_{min}$	422.33	911.29	410.65	1117.33	1965.48	1118.77
	$L_{max}$	740.25	27,719.03	853.83	1634.17	28,446.14	1790.57
	$\sigma_{L}$	40.93	7594.71	47.63	87.27	7536.50	83.89
**D (ms)**	$\bar{D}$	1998.00	2236.19	2000.01	2008.49	2246.74	1999.50
	$D_{min}$	1664.95	513.48	1588.44	1698.59	703.07	1348.73
	$D_{max}$	2277.76	3559.76	2439.89	2516.58	4259.41	2606.50
**J (ms)**		65.73	505.63	72.17	117.32	546.33	119.32
**PDR (%)**		100%	89.6%	100%	100%	88.8%	100%

## Data Availability

The raw data supporting the conclusions of this article will be made available by the corresponding author on request.

## Abbreviations

The following abbreviations are used in this manuscript:

5G NR	5th generation new radio
AI	Artificial Intelligence
AMQP	advanced message queuing protocol
API	application programming interface
BLE	Bluetooth low energy
BW	bandwidth
CCTV	closed circuit television
CI/CD	continuous integration/continuous delivery
CNCF	Cloud Native Computing Foundation
CoAP	constrained application protocol
CSV	comma-separated values
DDoS	distributed denial of service
DDS	data distribution service
DevOps	development and operations
EMF	Eclipse Modeling Framework
EU	European Union
GDPR	General Data Protection Regulation
gRPC	Google remote procedure call
GUI	graphical user interface
GW	gateway
HSM	hardware security module
HTTP	hypertext transfer protocol
IoT	Internet of Things
IP	Internet protocol
IT	information technology
JSON	JavaScript object notation
KMS	Key Management System
LPWAN	low-power wide area network
MAC	medium-access control (layer)
MCU	microcontroller unit
MEC	multi-access edge computing
ML	machine learning
MoCC	model of computation and communication
MQTT	message queuing telemetry transport
NB-IoT	narrowband Internet of Things
OCF	Open Connectivity Foundation
OS	operating system
OSGi	Open Services Gateway Initiative
PaaS	platform-as-a-service
PDR	packet delivery ratio
PHY	physical (layer)
PoC	proof of concept
QoS	quality of service
RAN	radio access network
REST	representational state transfer
RL	reinforcement learning
RPC	remote procedure call
SAREF	Smart Appliances REFerence
SDF	synchronous data flow
SDN	software defined radio
SLA	service level agreement
SSL	secure sockets layer
SSO	single sign-on
TCP	transmission control protocol
TEE	trusted execution environment
TPM	trusted platform module
UDP	user datagram protocol
UI	user interface
VM	virtual machine
WPAN	wireless personal area network
XMPP	extensible messaging and presence protocol
